# Gut microbiota as predictors of the occurrence of high on-treatment platelet reactivity in acute ischemic stroke patients

**DOI:** 10.3389/fcimb.2023.1257317

**Published:** 2024-01-04

**Authors:** Zhenzhen Lou, Huiying Ouyang, Guixian Chen, Xiaojun Li, Haoxuan Chen, Yibo Zhan, Lilin Peng, Chenghao Du, Zequan Zheng, Longlong Wen, Haoyou Xu, Min Zhao, Yuanqi Zhao

**Affiliations:** ^1^ The Second Affiliated Hospital of Guangzhou University of Chinese Medicine, Guangdong Provincial Hospital of Chinese Medicine, Guangzhou, China; ^2^ The Second Clinical College of Guangzhou University of Chinese Medicine, Guangzhou, China

**Keywords:** acute ischemic stroke, high on-treatment platelet reactivity, gut microbiota, 16S rRNA sequencing, random forest model

## Abstract

**Introduction:**

In this study, we aimed to investigate the association between gut microbiota and high on-treatment platelet reactivity (HTPR) in patients with acute ischemic stroke (AIS).

**Methods:**

We enrolled a total of 48 AIS patients, including 19 HTPR patients and 29 non-high on-treatment platelet reactivity (NHTPR) patients, along with 10 healthy controls. Clinical and laboratory data, as well as stool samples, were collected from all participants. The composition and function of gut microbiota were assessed using 16S rRNA sequencing. Differences in the gut microbiota between the two groups were analyzed, and a diagnostic model based on the gut microbiota was established using random forest model.

**Results:**

HTPR patients exhibited a decreased microbial richness compared to NHTPR patients. Additionally, the relative abundance of *unidentified_Clostridia* and *Ralstonia* was lower in HTPR patients. Significant differences in biological functions, such as toxoplasmosis, were observed between the two groups. The combination of *Ralstonia, unidentified-Clostridia, Mailhella, Anaerofustis*, and *Aggregatibacter* showed excellent predictive ability for HTPR occurrence (AUC=0.896). When comparing AIS patients with healthy controls, alterations in the microbiota structure were observed in AIS patients, with imbalances in short-chain fatty acid-producing bacteria and pathogenic bacteria. Significant differences in biological functions, such as oxidative phosphorylation, were noted between the two groups. The combination of *Alloprevotella, Terrisporobacter, Streptococcus, Proteus*, and *unidentified_Bacteria* exhibited strong predictive power for AIS occurrence (AUC=0.994).

**Conclusions:**

This study is the first to uncover the microbial characteristics of HTPR in AIS patients and demonstrate the predictive potential of specific bacterial combinations for HTPR occurrence.

## 1 Introduction

Stroke has emerged as the leading cause of disability and mortality among Chinese adults (Hu et al., 2020). In recent years, the incidence and prevalence of acute ischemic stroke (AIS) have continued to rise, even after standardizing for age (Wang et al., 2022). Antiplatelet therapy, particularly aspirin and clopidogrel, is fundamental in secondary prevention of AIS. However, despite persistent adherence to guideline-based secondary stroke prevention, approximately 8.3% of AIS patients still experience recurrent stroke within 12 months (Pan et al., 2021). The reason may be that antiplatelet drugs do not play the expected effect, which is known as high on-treatment platelet reactivity (HTPR) (Fiolaki et al., 2017; Shah et al., 2022). HTPR has been significantly associated with unfavorable outcomes following AIS (Zhou et al., 2022). Therefore, it is important to identify factors associated with HTPR in AIS patients.

Previous study revealed that trimethylamine N-oxide (TMAO), a gut microbial metabolite, increased platelet reactivity by promoting intracellular Ca2+ release, thereby contributing to thrombosis in mice; and specific gut microbial taxa were related to thrombosis in mice, for example, microbial taxa characterized by a high choline diet were significantly positively associated with enhanced thrombosis (Zhu et al., 2016), which highlighted the significant role of gut microbiota in the thrombotic process. In patients with ST-segment elevation myocardial infarction (STEMI) receiving ticagrelor treatment, it was observed that HTPR patients exhibited higher microbial richness and diversity compared to non-high on-treatment platelet reactivity (NHTPR) patients, and fecal transplantation experiments demonstrated that gut microbiota dysbiosis might be an important mechanism for the ticagrelor of HTPR (Zhang et al., 2022). Nevertheless, no previous studies have reported the association between gut microbiota and HTPR in AIS patients, leaving the microbial characteristics of HTPR in AIS patients largely unexplored.

Hence, this study aimed to investigate the microbial characteristics of HTPR in AIS patients, elucidate the potential functions of the gut microbiota in HTPR patients with AIS, and establish a predictive model for HTPR based on the gut microbiota.

## 2 Materials and methods

### 2.1 Study patients and data collection

All participants, including AIS patients and healthy controls, were recruited from the Brain Center of Guangdong Provincial Hospital of Traditional Chinese Medicine between February 2022 and January 2023. AIS diagnosis was made based on the Chinese guidelines for the diagnosis and treatment of acute ischemic stroke 2018 (Neurology and Society, 2018). The inclusion criteria for AIS patients were as follows: (1) age ≥ 18 years, (2) administration of clopidogrel (300 mg/day) or aspirin (300 mg/day), or a combination of clopidogrel (300 mg/day) and aspirin (300 mg/day) for at least 12 hours prior to the platelet function test; or administration of aspirin (100 mg/day) or clopidogrel (75 mg/day), or a combination of clopidogrel (75 mg/day) and aspirin (100 mg/day) for at least 5 days before the platelet function test, (3) informed consent obtained. The exclusion criteria for AIS patients were as follows: (1) cardiogenic stroke, (2) use of drugs that affect platelet aggregation, such as traditional non-steroidal antiplatelet drugs, ticlopidine, warfarin, heparin, and low molecular weight heparin, within 1 week before enrollment; use of antibiotics, probiotics, or glucocorticoids within 3 months before enrollment, (3) platelet count < 100×10^9/L or > 450×10^9/L, (4) myelodysplastic syndromes, (5) history of bleeding within the last 3 months or major surgical procedures within the last 4 weeks, (6) history of gastrointestinal diseases such as inflammatory bowel disease or gastrointestinal tumor; or use of alcohol, laxatives, or a history of drug abuse, (7) contraindication to clopidogrel or aspirin, (8) liver or kidney function damage (≥ three times the upper normal limit), (9) pregnancy or lactation, (10) participation in other clinical trials within the last 3 months. The inclusion criteria for healthy controls were as follows: (1) age ≥ 18 years, (2) no history of acute cardio-cerebrovascular events or myocardial infarction within the past 6 months, (3) informed consent obtained. The exclusion criteria for healthy controls were as follows: (1) use of antibiotics, probiotics, or glucocorticoids within 3 months before enrollment, (2) history of gastrointestinal diseases such as inflammatory bowel disease or gastrointestinal tumor, (3) use of alcohol, laxatives, or a history of drug abuse, (4) pregnancy or lactation, (5) participation in other clinical trials within the last 3 months.

Clinical data, including age, gender, medical history, medication history, as well as laboratory data, such as blood lipid levels and blood glucose levels, were collected.

### 2.2 Sample size calculation

The sample size was calculated using PASS software. According to the previous study, we used PD whole tree diversity index as the evaluating index. Based on Zhang’s study (Zhang et al., 2022), the average values of PD whole tree diversity index in HTPR and NHTPR groups were approximately16 and 13, respectively. The sample sizes of the HRPR and NHTPR groups should be 20 and 30, respectively (Power=0.8, significance level=0.05, mean difference=3, SD=3, allocation ratio=1.5).

### 2.3 Platelet function assessment

Peripheral venous blood samples were collected in 3.2% trisodium citrate for platelet function assessment. Two methods, namely light transmission aggregometry (LTA) and thromboelastography (TEG), were used in this study to evaluate platelet function.

LTA: The blood samples were centrifuged at 150 g/min for 10 minutes at room temperature to obtain platelet-rich plasma (PRP), while platelet-poor plasma (PPP) was obtained by centrifuging PRP at 3000 g/min for 10 minutes. ADP (150 μmol/L) or arachidonic acid (5 mg/mL) was added to the PRP as inducers. The decrease in plasma turbidity and increase in light transmittance were measured. The platelet aggregator recorded the dynamic change in light transmittance and generated the platelet aggregation curve. In this study, a maximum platelet aggregation rate of ≥50% when ADP (150 μmol/L) was used as the inducer was defined as HTPR on clopidogrel (Jia et al., 2021). Similarly, a maximum platelet aggregation rate of ≥20% when arachidonic acid (5 mg/mL) was used as the inducer was defined as HTPR on aspirin (Jia et al., 2021).

TEG: According to manufacturers’ instructions, TEG used four channels to assess platelet function. The maximum amplitude (MA), which represents the maximal clot strength, was used to evaluate the maximum platelet function stimulated by the inducer. MA_ADP_ represented the ADP-induced maximal clot strength, MA_AA_ represented the arachidonic acid-induced maximal clot strength, MA_fibrin_ represented the maximal clot strength induced by fibrin, and MA_thrombin_ represented the maximal clot strength induced by thrombin. The percentage of platelet inhibition induced by ADP/AA was calculated using the formula: ADP/AA%= [(MA_thrombin_ – MA_ADP/AA_)/(MA_thrombin_ - MA_fibrin_)] ×100%. In this study, ADP% < 30% or MA_ADP_ > 47 mm was considered as HTPR on clopidogrel, while AA% < 50% was considered as HTPR on aspirin (Yang et al., 2020).

AIS patients exhibiting HTPR on clopidogrel or aspirin were classified as the HTPR group, while AIS patients without HTPR were classified as the NHTPR group.

### 2.4 Stool sample collection, DNA extraction and 16S RNA gene amplicon and sequencing

Stool samples (200 mg) were collected and stored in the Fecal Microbial Collection and Preservation Kit (ML-001A, Shenzhen Dayun Gene Technology Co., Ltd.) and saved in an -80°C refrigerator within 3 days (Chen et al., 2022; Luo et al., 2022). Total DNA was extracted following the manufacturer’s instructions using CTAB method. To further ensure the quality of extracted total genomic DNA, the integrity and concentration of the extract were evaluated using 1% agarose gel electrophoresis. The 16S V4 region of the bacterial 16S rRNA gene was amplified using specific primers (515F - 806R). Subsequently, the amplified products were evaluated by 2% agarose gel electrophoresis. PCR products was mixed in equidensity ratios. Then, mixture PCR products was purified with Qiagen Gel Extraction Kit (Qiagen, Germany). Sequencing libraries were generated using the TruSeq DNA PCR-Free Sample Preparation Kit (Illumina, USA) and index codes were added. The library quality was assessed on the Qubit@ 2.0 Fluorometer (Thermo Scientific) and Agilent Bioanalyzer 2100 system. At last, the library was sequenced on an Illumina NovaSeq platform and 250 bp paired-end reads were generated. Raw Tags were filtered by Qiime (Version 1.9.1, http://qiime.org/scripts/split_libraries_fastq.html) (Caporaso et al., 2010). High-quality sequences were clustered into Operational Taxonomic Units (OTUs) with similarity≥97% by Uparse software (Uparse v7.0.1001, http://drive5.com/uparse/) (Edgar, 2013). Then OTUs were classified into the statistics of each level (phylum, class, order, family, genus, species), and eventually an OTU table was created. Qiime (Version 1.9.1) was used to analyze Alpha diversity and Beta diversity. Linear discriminant analysis (LDA) effect size (LEfSe) was used to find taxa with significant differences (LDA > 2.0).

### 2.5 Statistical analyses

Statistical analysis was performed using SPSS 18.0 software (Statistical Package for Social Sciences, Chicago, USA). Categorical variables were presented as numbers and percentages and analyzed using the chi-square test. Continuous variables with a normal distribution were expressed as mean ± standard deviation (SD) and analyzed using the Student’s t-test. Continuous variables without a normal distribution were described as median (interquartile range (IQR)) and analyzed using the non-parametric Wilcoxon test. A generalized linear model (GLM) was employed to model the microbiota that was significantly different between the two groups after controlling for possible confounding factors. Spearman correlation analysis was used to identify associations between genera with significant differences in LEfSe analysis and pathways with significant differences in the predictive function analysis.

## 3 Results

### 3.1 Baseline characteristics

**Figure 1:**
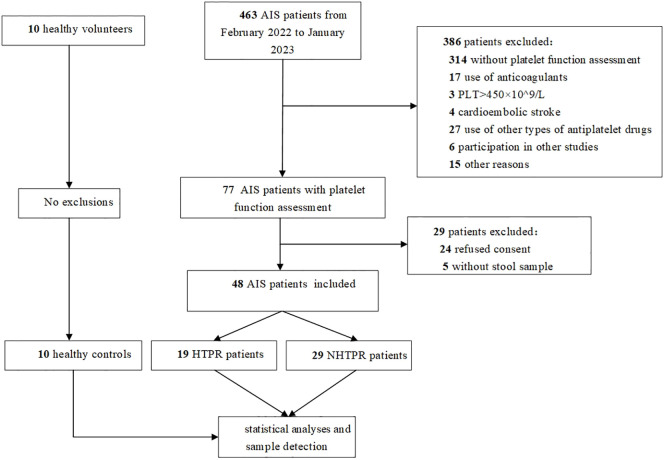
Flow chart. AIS: acute ischemic stroke, PLT, platelet count; HTPR, high on-treatment platelet reactivity; NHTPR, non-high on-treatment platelet reactivity.

A total of 463 patients were initially screened, there were 53 AIS patients signing informed consent after strict screening according to inclusion and exclusion criteria, 5 AIS patients without stool sample were excluded, and ultimately, 48 AIS patients were included in the study which were divided into 19 HTPR patients and 29 NHTPR patients. Additionally, 10 healthy controls were enrolled. The flow chart illustrating the participant selection process is presented in **Figure 1**. **Table 1** displays the baseline characteristics of the HTPR group and NHTPR group. The NHTPR group had a higher median BMI compared to the HTPR group (24.22 *vs.* 21.59, *P*=0.045). However, no significant differences were observed in other baseline data between the HTPR and NHTPR groups. **Table 2** presents the baseline characteristics of AIS patients and healthy controls. AIS patients were older (65.92 ± 10.29 *vs.* 53.00 ± 4.00, *P*<0.001) and had higher blood sugar levels (7.01 *vs.* 5.17, *P*=0.018) compared to the healthy controls. No statistically significant differences in other baseline data were observed between the AIS and healthy control groups.

**Table 1:** Baseline characteristics between the HTPR and NHTPR groups.

Parameter	N	HTPR group(n=19)	NHTPR group(n=29)	*P*
Male, n, (%)	48	12(63.2)	21(72.4)	0.499
Age, y, mean ± SD	48	67.21 ± 9.07	65.07 ± 11.08	0.486
BMI, kg/m^2^, M (P_25_, P_75_)	48	21.59(20.69,23.20)	24.22(22.04,26.67)	0.045
Smoking, n, (%)	48	8(42.1)	13(44.8)	0.853
Drinking, n, (%)	48	7(36.8)	7(24.1)	0.344
Medical history, n, (%)				
Cerebrovascular disease	48	5(26.3)	6(20.7)	0.732
Diabetes	48	7(36.8)	10(34.5)	0.867
Hypertension	48	14(73.7)	21(72.4)	0.923
Hyperlipemia	48	3(15.8)	3(10.3)	0.669
CardiovascuIar disease	48	3(15.8)	7(24.1)	0.719
Medication history, n, (%)				
Hypolipidemic drug	48	16(84.2)	27(93.1)	0.372
Hypoglycemic drug	48	7(36.8)	9(31.0)	0.676
PPIs	48	12(63.2)	21(72.4)	0.499
Laboratory data, mean ± SD/M (P_25_, P_75_)				
PLT, × 10^9/L	48	232.50(212.75,343.50)	240.00(202.00,334.00)	0.411
TG, mmol/L	45	1.39(1.08,2.17)	1.71(1.21,2.01)	0.487
TC, mmol/L	45	4.47 ± 1.24	4.81 ± 0.93	0.299
HDL-C, mmol/L	45	1.00(0.86,1.21)	1.09(0.98,1.35)	0.098
LDL-C, mmol/L	45	2.80 ± 1.11	3.15 ± 0.85	0.242
BG, mmol/L	48	6.66(5.44,8.56)	7.03(5.35,8.62)	0.808

BMI, body mass index; PPIs, proton pump inhibitors; PLT, platelet count; TG, triglyceride; TC, total cholesterol; HDL-C, high-density lipoprotein-cholesterol; LDL-C, low-density lipoprotein-cholesterol; BG, blood glucose.

**Table 2:** Baseline characteristics between the AIS and healthy control groups.

Parameter	N	AIS group (n=48)	healthy control group (n=10)	*P*
Male, n, (%)	58	33(68.8)	5(50.0)	0.290
Age, y, mean ± SD	58	65.92 ± 10.29	53.00 ± 4.00	<0.001
BMI, kg/m^2^, M (P_25_, P_75_)	55	22.94 (21.17,26.38)	24.23 (21.40,25.35)	0.750
Smoking, n, (%)	58	21(43.8)	2(20.0)	0.287
Drinking, n, (%)	58	14(29.2)	3(30.0)	1.000
Medical history, n, (%)				
Cerebrovascular disease	58	11(22.9)	0(0)	0.182
CardiovascuIar disease	58	10(20.8)	0(0)	0.184
Laboratory data, mean ± SD/M (P_25_, P_75_)				
PLT, × 10^9/L	58	240.0(207.5,338.5)	224.0(202.5,261.0)	0.393
TG, mmol/L	55	1.64(1.17,2.01)	1.69(1.31,2.09)	0.600
TC, mmol/L	55	4.67 ± 1.07	4.24 ± 0.66	0.222
HDL-C, mmol/L	55	1.08(0.96,1.33)	1.12(0.97,1.33)	0.662
LDL-C, mmol/L	55	3.01 ± 0.97	2.89 ± 0.47	0.556
BG, mmol/L	58	7.01(5.41,8.56)	5.17(4.69,6.22)	0.018

BMI, body mass index; PLT, platelet count; TG, triglyceride; TC, total cholesterol; HDL-C, high-density lipoprotein-cholesterol; LDL-C, low-density lipoprotein-cholesterol; BG, blood glucose.

### 3.2 Comparison of gut microbiota diversity

**Figure 2:**
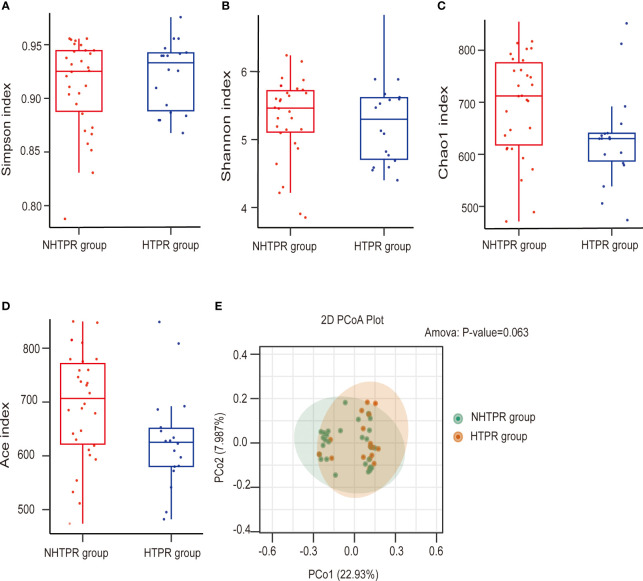
Comparison of the microbial communities of the HTPR and NHTPR groups. Box plots depict differences in the gut microbiota diversity indices between the HTPR and NHTPR groups according to the Simpson index **(A)**, Shannon index **(B)**, Chao1 index **(C)**, and Ace index **(D)**; each box plot represents the median, interquartile range, minimum, and maximum values. PCoA shows the gut microbiota composition in the HTPR and NHTPR groups **(E)**; PCoA, principal coordinates analysis.

To assess microbial richness, α-diversity analyses were performed using the Shannon index, Simpson index, Chao1 index, and Ace index. β-diversity analyses, employing principal coordinate analysis (PCoA), were conducted to compare differences in microbiota structure. The results indicated no statistical differences in the Simpson index and Shannon index between the HTPR and NHTPR groups (Wilcoxon rank-sum test, *P*=0.558 for the Simpson index, **Figure 2A**; *P*=0.476 for the Shannon index, **Figure 2B**). However, the Chao1 index and Ace index of the NHTPR group were significantly higher than those of the HTPR group (Wilcoxon rank-sum test, *P*=0.019 for the Chao1 index, **Figure 2C**; *P*=0.022 for the Ace index, **Figure 2D**). These findings suggested that compared to the NHTPR group, the HTPR group exhibited decreased microbial richness. PCoA analysis indicated a similarity in microbial structures between the HTPR and NHTPR groups (*P*=0.063, **Figure 2E**).

**Figure 3:**
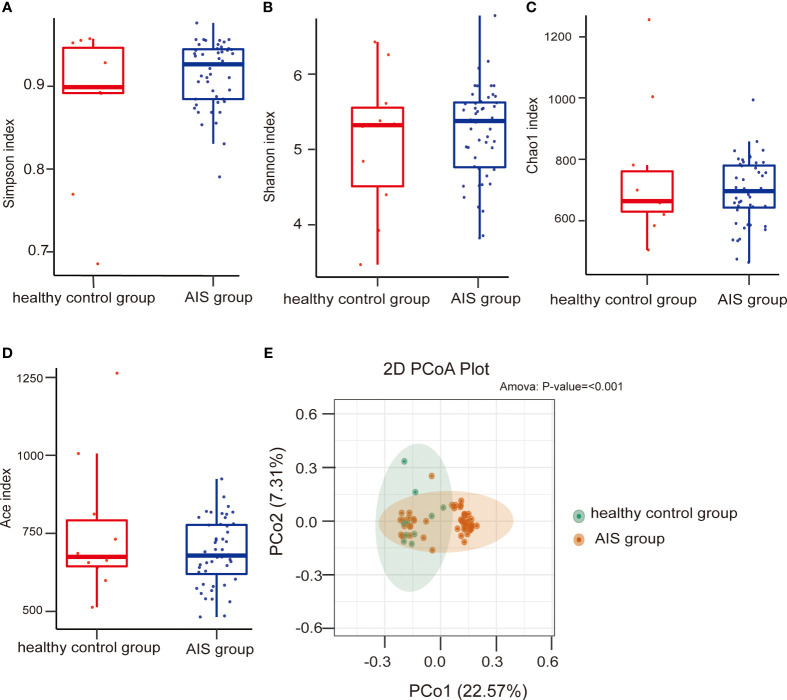
Comparison of the microbial communities of the AIS and healthy control groups. Box plots depict differences in the gut microbiota diversity indices between the AIS and healthy control groups according to the Simpson index **(A)**, Shannon index **(B)**, Chao1 index **(C)**, and Ace index **(D)**; each box plot represents the median, interquartile range, minimum, and maximum values. PCoA shows the gut microbiota composition in the AIS and healthy control groups **(E)**; PCoA, principal coordinates analysis.

No statistically significant differences were observed in the Simpson index, Shannon index, Chao1 index, and Ace index between the AIS and healthy control groups (Wilcoxon rank-sum test, *P*=0.628 for the Simpson index, **Figure 3A**; *P*=0.658 for the Shannon index, **Figure 3B**; *P*=0.879 for the Chao1 index, **Figure 3C**; *P*=0.723 for the Ace index, **Figure 3D**), indicating similar microbial richness between the AIS and healthy control groups. PCoA analysis demonstrated a significant difference in microbial structure between the AIS and healthy control groups (*P*<0.001, **Figure 3E**).

### 3.3 LEfSe analysis

**Figure 4:**
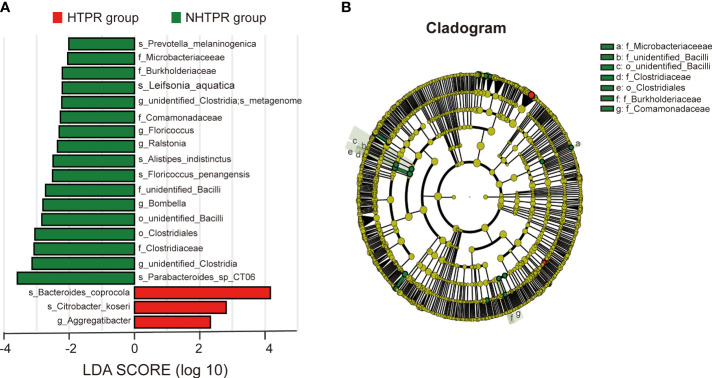
LEfSe analysis revealed taxonomic differences of gut microbiota in the HTPR and NHTPR groups. **(A)** The bar plots showed significant differences in gut microbiota between the HTPR group and NHTPR group. The LDA scores (log10) >2 and p < 0.05 are listed. **(B)** Cladogram indicated the phylogenetic distribution of the gut microbiota associated with the HTPR and NHTPR groups. Red indicates communities with increased relative abundance in the HTPR group, green indicates communities with increased relative abundance in the NHTPR group, and yellow nodes indicate microbial communities that do not play an important role in both groups.

**Figure 5:**
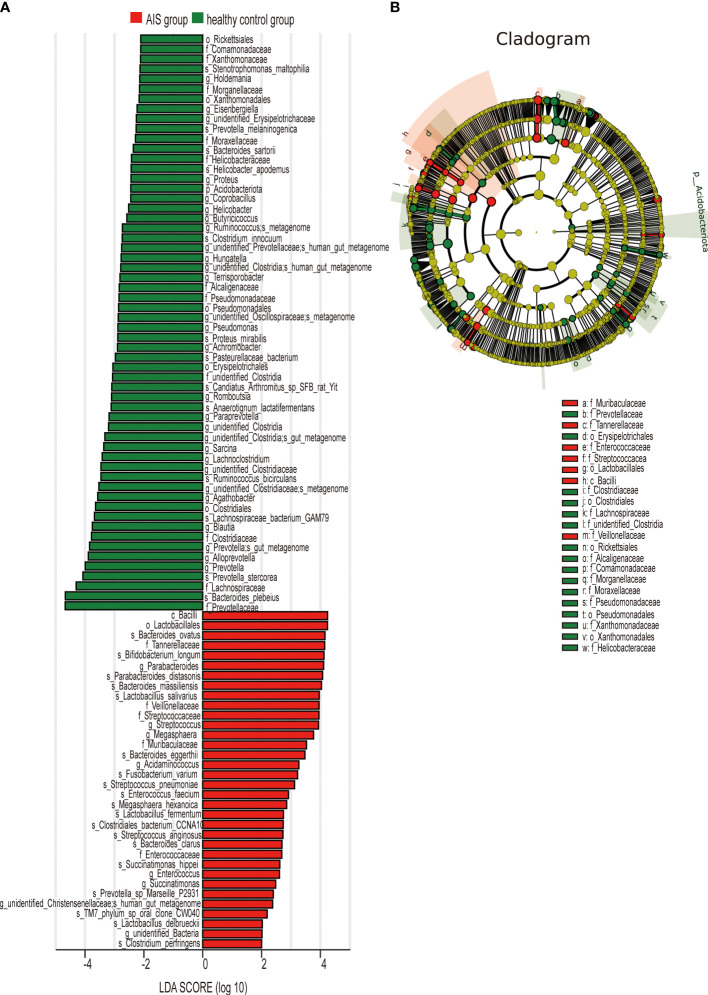
LEfSe analysis revealed taxonomic differences of gut microbiota in the AIS and healthy control groups. **(A)** The bar plots showed significant differences in gut microbiota between the AIS group and healthy control group. The LDA scores (log10) >2 and p < 0.05 are listed. **(B)** Cladogram indicated the phylogenetic distribution of the gut microbiota associated with the AIS and healthy control groups. Red indicates communities with increased relative abundance in the AIS group, green indicates communities with increased relative abundance in the healthy control group, and yellow nodes indicate microbial communities that do not play an important role in both groups.

LEfSe analysis was employed to compare the gut microbiota composition among different groups. The LDA score cutoff of 2.0 was applied to identify taxonomic differences of significance between the groups. The distribution of LDA scores is depicted in **Figures 4A**, **5A**, while the cladogram based on the LEfSe method is presented in **Figure 4B** and **Figure 5B**. Here, we considered the differences at the genus level. **Figure 4A** reveals five bacterial species at the genus level that differ between HTPR patients and NHTPR patients, namely *Floricoccus, Ralstonia*, *Bombella*, *unidentified-Clostridia*, *Aggregatibacter*. After controlling for potential confounding factors (such as BMI), GLM was employed to assess the significant differences in the identified genera between the two groups. Notably, *unidentified_Clostridia* and *Ralstonia* exhibited substantial differences (*P* < 0.05), suggesting that these genera were associated with HTPR (**Table 3**).

**Table 3:** GLM based on differences at the genus level between the HTPR and NHTPR groups.

Names	HTPR group		NHTPR group		*b*	95%CI	*P*
	Mean	SD	Mean	SD			
G_unidentified_Clostridia	1.18e-03	6.49e-04	3.51e-03	7.10e-03	988.86	290.16~1979.90	2.23e-02
G_Ralstonia	1.01e-06	4.42e-06	3.02e-04	1.51e-03	127689.88	39166.76~284778.65	2.99e-02

The b-value is the correlation coefficient. GLM, general linear model; SD, standard deviation; CI, confidence interval.

**Figure 5A** displays 28 bacterial species at the genus level that demonstrate differences between the AIS and healthy control groups. Following adjustments for potential confounders (such as age and blood glucose), the primary distinctions between the two groups were linked to *Parabacteroides*, *Streptococcus*, *Blautia*, *Lachnoclostridium*, and *unidentified_Bacteria* (*P* < 0.05), indicating that these genera were related to AIS (**Table 4**).

**Table 4:** GLM based on differences at the genus level between the healthy control and AIS groups.

Names	healthy control group		AIS group		*b*	95%CI	*P*
	Mean	SD	Mean	SD			
g_Parabacteroides	1.16e-02	8.54e-03	3.69e-02	6.22e-02	-175.99	-341.71~-61.49	1.07e-02
g_Streptococcus	3.66e-03	3.62e-03	2.27e-02	2.90e-02	-391.06	-808.23~-143.47	1.69e-02
g_Blautia	2.08e-02	1.41e-02	1.18e-02	7.50e-03	228.03	63.54~479.61	2.65e-02
g_Lachnoclostridium	1.54e-02	7.97e-03	1.09e-02	9.33e-03	122.05	10.52~251.49	3.77e-02
g_unidentified_Bacteria	4.04e-05	5.17e-05	2.22e-04	2.48e-02	-18494.07	-40482.38~-4423.29	3.65e-02

The b-value is the correlation coefficient. GLM, general linear model; SD, standard deviation; CI, confidence interval.

### 3.4 Predictive function analysis

**Figure 6:**
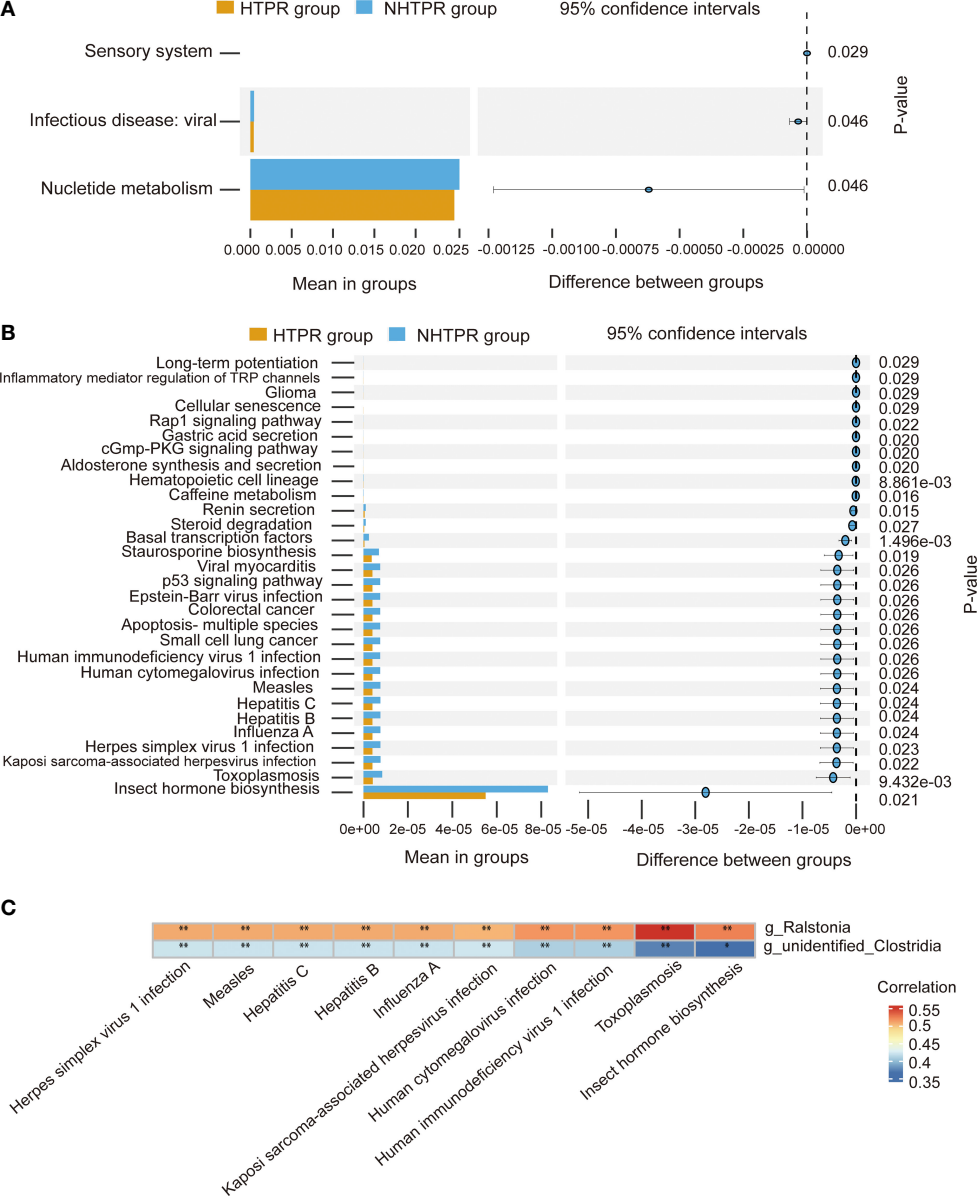
Functional predictions of the gut microbiota and heatmap of Spearman’ s correlation analysis in the HTPR and NHTPR groups. **(A)** The main metabolic pathways with significant differences between the HTPR and NHTPR groups at level 2. **(B)** The top 30 metabolic pathways with significant differences between the HTPR and NHTPR groups at level 3. **(C)** Heatmap of Spearman’ s correlation analysis between the pathways with significant differences and the top 10 relative abundance at level 3 and the genera with significant differences. *p < 0.05, **p < 0.01.

To explore the functional characteristics of the gut microbiota in HTPR patients, we employed the PICRUSt2 analysis method, utilizing the Kyoto Encyclopedia of Genes and Genomes (KEGG) database and 16S rRNA gene sequence data for functional prediction. **Figure 6A** presents the main significantly different metabolic pathways at level 2 between the HTPR and NHTPR groups. Compared to the NHTPR group, the HTPR group exhibited reduced relative abundance in nucleotide metabolism, infectious disease-viral, and sensory system pathways. At level 3, there were 47 metabolic pathways displaying differences between the HTPR and NHTPR groups. **Figure 6B** illustrates the top 30 metabolic pathways with significant differences, all of which exhibited lower relative abundance in the HTPR group compared to the NHTPR group. Notably, insect hormone biosynthesis displayed the highest relative abundance, followed by toxoplasmosis. Spearman’s correlation analysis was performed between the pathways with significant differences, the top 10 relative abundance at level 3, and the genera exhibiting significant differences. *Ralstonia* demonstrated a moderate correlation with the above 10 pathways, with the highest correlation observed with toxoplasmosis. On the other hand, *unidentified_Clostridia* displayed a weak correlation with the above 10 pathways, with the lowest correlation observed with insect hormone biosynthesis (**Figure 6C**).

**Figure 7:**
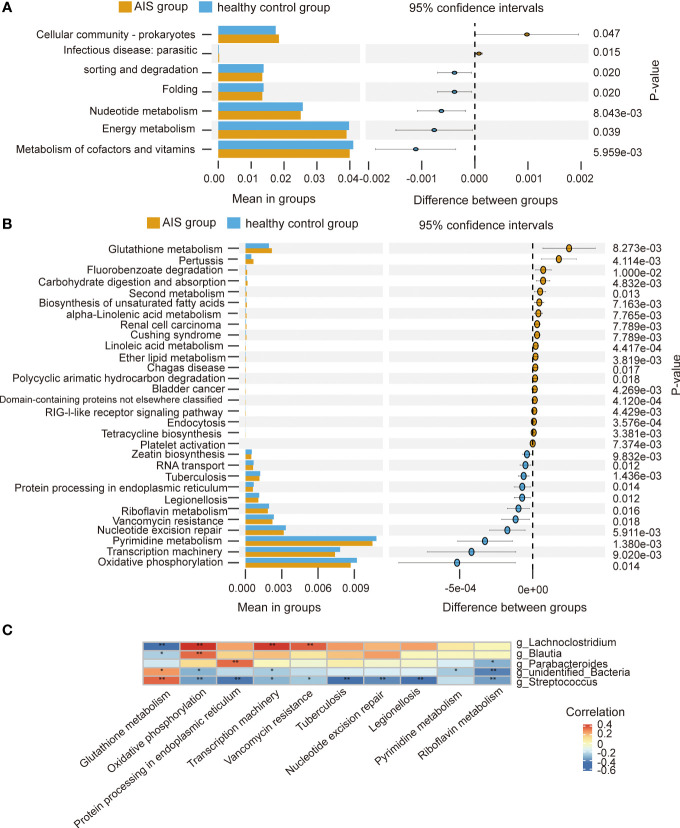
Functional predictions of the gut microbiota and heatmap of Spearman’ s correlation analysis in the AIS and healthy control groups. **(A)** The main metabolic pathways with significant differences between the AIS and healthy control groups at level 2. **(B)** The top 30 metabolic pathways with significant differences between the AIS and healthy control groups at level 3. **(C)** Heatmap of Spearman’ s correlation analysis between the pathways with significant differences and the top 10 relative abundance at level 3 and the genera with significant differences. *p < 0.05, **p < 0.01.

At level 2, there were 7 metabolic pathways exhibiting significant differences between the AIS and healthy control groups (**Figure 7A**). Among these, metabolism of cofactors and vitamins displayed the highest relative abundance, followed by energy metabolism. At level 3, there were 69 metabolic pathways showing differences between the AIS and healthy control groups. **Figure 7B** depicts the top 30 metabolic pathways with significant differences, with pyrimidine metabolism displaying the highest relative abundance, followed by oxidative phosphorylation. Spearman’s correlation analysis was conducted between the pathways with significant differences and the top 10 relative abundance at level 3 and the genera displaying significant differences. **Figure 7C** reveals that *Lachnoclostridium* exhibits a positive correlation with oxidative phosphorylation, transcription machinery, and vancomycin resistance, and a negative correlation with glutathione metabolism; similarly, *Blautia* demonstrats a positive correlation with oxidative phosphorylation and a negative correlation with glutathione metabolism; *Parabacteroides* displays a positive correlation with protein processing in the endoplasmic reticulum and a negative correlation with riboflavin metabolism; *unidentified_Bacteria* exhibits a positive correlation with glutathione metabolism and a negative correlation with riboflavin metabolism, oxidative phosphorylation, transcription machinery, and pyrimidine metabolism; *Streptococcus* displays a positive correlation with glutathione metabolism and a negative correlation with protein processing in the endoplasmic reticulum, oxidative phosphorylation, transcription machinery, nucleotide excision repair, vancomycin resistance, riboflavin metabolism, tuberculosis, and legionellosis.

### 3.5 Random forest predictive models

**Figure 8:**
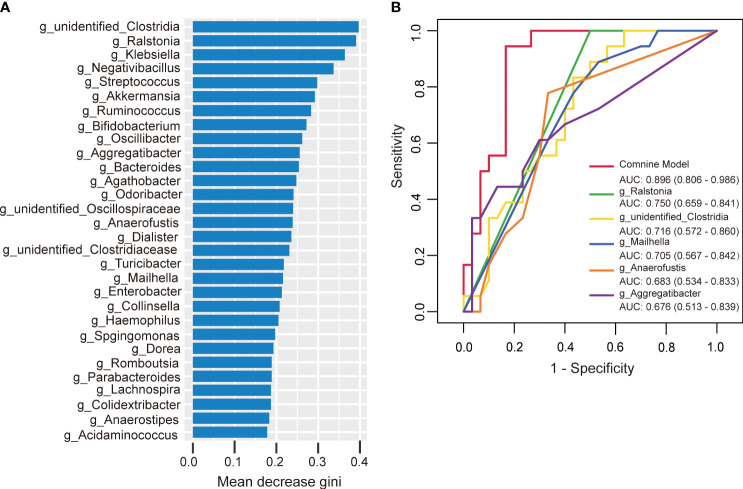
The predictive model based on genus-level abundance taxa using a random forest model in the HTPR group. **(A)** The relative importance of each genus was assessed by the mean decrease gini. **(B)** ROC curve constructed by random forest in gut microbiota. AUC is shown in the bottom right corner of the figure with 95% confidence intervals in parentheses. ROC, receiver operating characteristic; AUC, area under the ROC curve.

To further investigate the potential of gut microbiota in diagnosing HTPR, we employed random forest to develop a prediction model using the bacterial species with significant importance at the genus level as input. The relative importance of each genus in the predictive model was evaluated using mean decrease Gini. Among the 30 genera analyzed, *unidentified-Clostridia* ranked first in terms of importance based on mean decrease Gini (**Figure 8A**). Notably, *Ralstonia, unidentified-Clostridia, Mailhella, Anaerofustis*, and *Aggregatibacter* showed significant associations with the occurrence of HTPR, with areas under the receiver operating characteristic (ROC) curve (AUC) of 0.750, 0.716, 0.705, 0.683, and 0.676, respectively. To enhance diagnostic accuracy, we constructed an ROC curve incorporating these five bacterial species, resulting in an AUC of 0.896 (95% confidence interval: 0.806–0.986) (**Figure 8B**). Hence, gut microbiota holds promise as a potential diagnostic tool for HTPR.

**Figure 9:**
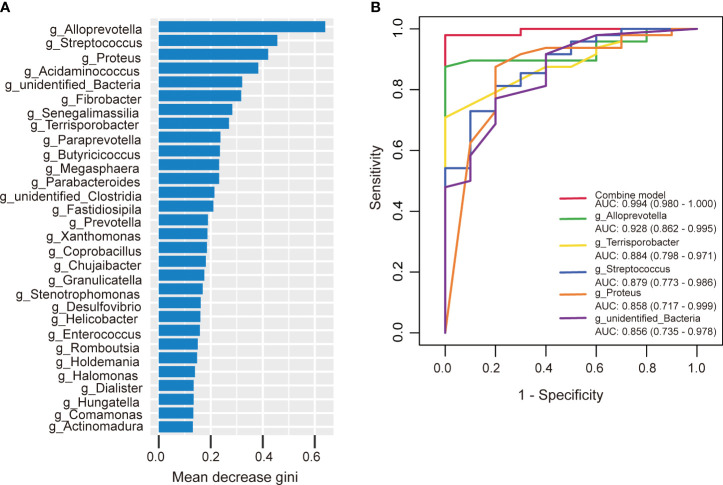
The predictive model based on genus- level abundance taxa using a random forest model in the AIS group. **(A)** The relative importance of each genus was assessed by the mean decrease gini. **(B)** ROC curve constructed by random forest in gut microbiota. AUC is shown in the bottom right corner of the figure with 95% confidence intervals in parentheses. ROC, receiver operating characteristic; AUC, area under the ROC curve.

Similarly, we developed a prediction model using random forest and the bacterial species with significant importance at the genus level to predict the occurrence of AIS. Among the 30 genera analyzed, *Alloprevotella* ranked first in terms of mean decrease Gini (**Figure 9A**). *Alloprevotella, Terrisporobacter, Streptococcus, Proteus*, and *unidentified_Bacteria* were significantly associated with the occurrence of AIS, exhibiting AUCs of 0.928, 0.884, 0.879, 0.858, and 0.856, respectively. To improve diagnostic performance, we constructed a ROC curve incorporating these five bacterial species, yielding an AUC of 0.994 (95% confidence interval: 0.980–1.000) (**Figure 9B**). These findings suggest that gut microbiota has the potential to serve as a valuable diagnostic tool for AIS.

## 4 Discussion

In our study, we observed alterations in the gut microbiota and functions in patients with HTPR and AIS. Specific combinations of bacterial genera demonstrated predictive potential for HTPR and AIS occurrence. Here are the key findings regarding the gut microbiota characteristics in HTPR and AIS patients: HTPR patients exhibited a decreased microbial richness compared to NHTPR patients; additionally, the relative abundance of *unidentified_Clostridia* and *Ralstonia* was lower in HTPR patients; significant differences in biological functions, such as toxoplasmosis, were observed between the two groups; the combination of *Ralstonia, unidentified-Clostridia, Mailhella, Anaerofustis*, and *Aggregatibacter* showed excellent predictive ability for HTPR occurrence; when comparing AIS patients with healthy controls, alterations in the microbiota structure were observed in AIS patients, with imbalances in short-chain fatty acid-producing bacteria and pathogenic bacteria; significant differences in biological functions, such as oxidative phosphorylation, were noted between the two groups; the combination of *Alloprevotella, Terrisporobacter, Streptococcus, Proteus*, and *unidentified_Bacteria* exhibited strong predictive power for AIS occurrence.

### 4.1 The microbial richness decreased and the bacterial taxonomic composition changed in HTPR patients

So far, there is only one study reported by Zhang X et al. investigating the relationship between gut microbiota and HTPR in patients with STEMI after ticagrelor treatment (Zhang et al., 2022). There is no published study on the relationship between the gut microbiota and HTPR in AIS patients. Zhang X et al. (Zhang et al., 2022) explored the mechanism of the poor response of ticagrelor in patients with STEMI and found that HTPR patients had increased richness and altered microbiota structure compared with NHTPR patients, while the results of our study showed that the microbial richness of the HTPR patients decreased compared with NHTPR patients, and the microbial structures between the HTPR and NHPTR groups were similar. As we know, the diversity of gut microbiota is affected by many factors such as age, disease, diet, and lifestyle (Lozupone et al., 2012), which may be the reason for the different results of the two studies.

Furthermore, we observed a decrease in the relative abundance of *unidentified_Clostridia* and *Ralstonia* in HTPR patients. *Unidentified_Clostridia* have been reported to inhibit the growth of harmful bacteria, promote the growth of beneficial bacteria, enhance immunity, and support nutrient digestion and absorption (Liu et al., 2021). Therefore, the decrease in *unidentified_Clostridia* could disrupt the balance between beneficial and harmful bacteria, which might have a negative effect on health. Moreover, the absence of *unidentified_Clostridia* might influence the digestion and absorption of antiplatelet drugs, potentially reducing their bioavailability and efficacy, which could contribute to the occurrence of HTPR. *Ralstonia*, a prevalent pro-inflammatory opportunistic pathogen, has been previously found to be more abundant in fecal samples and sigmoid mucosa of Parkinson’s disease patients compared to controls (Keshavarzian et al., 2015). However, the role of *Ralstonia* in HTPR patients remains unclear and further investigations are warranted to determine its significance.

Functional predictions using PICRUSt2 analysis revealed differences in biological functions, such as toxoplasmosis, between the HTPR and NHTPR groups. Spearman’s correlation analyses indicated associations between *Ralstonia* and *unidentified_Clostridia* and specific pathways at level 3. For example, *Ralstonia* showed a moderate correlation with toxoplasmosis, while *unidentified_Clostridia* exhibited a weak correlation with insect hormone biosynthesis. These findings suggest that gut microbiota may influence the occurrence and progression of HTPR through these specific pathways. However, further studies are required to elucidate the potential mechanisms by which *unidentified_Clostridia* and *Ralstonia* impact HTPR through these pathways.

### 4.2 The combination of special bacteria could predict the occurrence of HTPR

In the study reported by Zhang et al. (Zhang et al., 2022), the occurrence of HTPR was predicted using random forest with the relative importance order of 21 genera as input, yielding the AUC of 0.807. Similarly, in our study, we successfully distinguished HTPR from NHTPR using specific bacterial genera. The AUCs of *Ralstonia, unidentified-Clostridia, Mailhella, Anaerofustis*, and *Aggregatibacter* were 0.750, 0.716, 0.705, 0.683, and 0.676, respectively, and the AUC of the combination of these five bacterial species was 0.896, highlighting the significant role of gut microbiota in HTPR diagnosis. However, it is worth noting that the diagnostic value of specific gut microbiota can vary across different studies. Therefore, large-scale multicenter, cross-ethnic, and cross-regional investigations are necessary to thoroughly evaluate the diagnostic potential of gut microbiota in HTPR.

### 4.3 The microbial structure and taxonomic composition changed in AIS patients

In our study, we did not observe a statistically significant difference in α-diversity analysis between the AIS and healthy control groups. However, β-diversity analysis revealed significant differences in the microbial structure between the AIS and healthy control groups. The relationship between α and β diversity of gut microbiota and AIS is currently a matter of debate. A previous study has shown that Chao1, observed species, and PD whole tree in IS or TIA group were significantly higher than those in control group, the Shannon index showed a similar trend, but the difference was not statistically significant, the β-diversity analysis showed that the microbial structure of IS or TIA group was significantly different from that of control group (Yin et al., 2015). Li et al. reported that there were no differences in Shannon index, Simpson index, ACE index, Chao1 index, and the bacterial community structures between ischemic stroke and healthy control groups (Li et al., 2019). The diversity of gut microbiota is influenced by various factors, which may be the reason for the differences in diversity analysis among different studies.

Moreover, we observed increased relative abundance of *Parabacteroides, Streptococcus*, and *unidentified_Bacteria*, and decreased relative abundance of *Lachnoclostridium* and *Blautia* in AIS patients. *Parabacteroides* is known to be an opportunistic pathogen, and its increased abundance has been associated with sepsis in ICU patients (Agudelo-Ochoa et al., 2020). *Streptococcus* can cause various infections, including impetigo, pharyngitis, and necrotizing fasciitis (Li et al., 2023). Mulla et al. has reported that *Streptococcus* may be related to interleukin-6 (IL6), tumor necrosis factor (TNF)-alpha, and other cytokines (Xiong et al., 2023). The increased abundance of *Parabacteroides* and *Streptococcus* suggests that opportunistic pathogens may proliferate in AIS patients, consistent with the pattern of post-stroke gut microbiota dysbiosis observed in previous studies (Yin et al., 2015; Sun et al., 2022). It has been reported that *unidentified_Bacteria* may be involved in acetic oxidation from butyrate (a short-chain fatty acid) metabolism (Yi et al., 2020). *Lachnoclostridium* and *Blautia* are known SCFA-producing bacteria (Kang et al., 2021; Zhao et al., 2021), and *Blautia* has anti-inflammatory effects and contributes to the recovery of intestinal mucosal injury (Zhao et al., 2021). The differential trends observed in these SCFA-producing bacteria among AIS patients warrant further investigation to elucidate their roles in AIS.

PICRUSt2 analysis revealed differences in biological functions between the AIS and healthy control groups. Spearman’s correlation analyses indicated that *Parabacteroides, Streptococcus*, *unidentified_Bacteria*, *Lachnoclostridium* and *Blautia* were associated with specific pathways at level 3. Future studies are required to validate the potential impact of these specific bacteria on AIS through these pathways.

### 4.4 The combination of special bacteria could predict the occurrence of AIS

In this study, the occurrence of AIS could be effectively diagnosed by special bacterial combination at the genus level. The AUCs of *Alloprevotella, Terrisporobacter, Streptococcus, Proteus, unidentified_Bacteria* were 0.928, 0.884, 0.879, 0.858, and 0.856, respectively. Remarkably, the combination of these five bacterial species yielded an impressive AUC of 0.994.

At present, there are some studies focusing on the gut microbiota of AIS or specific AIS populations, such as the study reported by Chen et al. (Chen et al., 2022). Chen’s study was to investigate the characteristic gut microbiota of poor outcome of AIS with hyperlipidemia (POAH) patients. They found that in POAH patients, the relative abundance of *Enterococcaceae* and *Enterococcus* were increased, and the relative abundance of *Lachnospiraceae*, *Faecalibacterium*, *Rothia* and *Butyricicoccus* were decreased. And they established a ROC model based on the combination of characteristic microbiota (*Unclassified-f-Lachnospiraceae*, *Enterococcus*, *Faecalibacterium*, *Lachnospiraceae-UCG-010*, and *norank-f-Lachnospiraceae*) to predict POAH (AUC = 0.694, 95% CI 0.618-0.770).

However, our study is the first to investigate the association between the gut microbiota and HTPR in AIS patients. In this study, we found that the relative abundance of *unidentified_Clostridia* and *Ralstonia* was lower in HTPR patients, and the combination of *Ralstonia, unidentified-Clostridia, Mailhella, Anaerofustis*, and *Aggregatibacter* showed excellent predictive ability for HTPR occurrence (AUC=0.896). And we observed increased relative abundance of *Parabacteroides, Streptococcus*, and *unidentified_Bacteria*, and decreased relative abundance of *Lachnoclostridium* and *Blautia* in AIS patients, the combination of *Alloprevotella, Terrisporobacter, Streptococcus, Proteus*, and *unidentified_Bacteria* exhibited strong predictive power for AIS occurrence (AUC=0.994).

In this study, several limitations should be mentioned. Firstly, this was a small-scale, single-center observational study, which may introduce inherent biases. Secondly, the use of 16S rRNA sequencing to analyze changes in gut microbiota may provide limited information compared to metagenomic sequencing, which offers more comprehensive insights into bacterial species and functions. Despite these limitations, our study provides novel insights into the microbial characteristics of HTPR patients with AIS, enhancing our understanding of the association between gut microbiota and HTPR in AIS patients. These findings also offer a theoretical basis for developing personalized medication strategies for HTPR from the perspective of gut microbiota.

## 5 Conclusion

In summary, this study is the first to uncover the microbial characteristics of HTPR in AIS patients and demonstrate the predictive potential of specific bacterial combinations for HTPR occurrence.

## Data availability statement

The data presented in the study are deposited in the National Center for Biotechnology Information repository, and the accession number is PRJNA995199.

## Ethics statement

This study was approved by the Ethics Committee of the Guangdong Provincial Hospital of Traditional Chinese Medicine (Grant No. YE2022-022-01) and conducted in accordance with the principles of the Declaration of Helsinki. Written informed consent was obtained from all participants.

## Author contributions

ZL: Data curation, Investigation, Methodology, Writing – original draft, Writing – review & editing. HO: Data curation, Writing – review & editing. GC: Data curation, Writing – review & editing. XL: Data curation, Writing – review & editing. HC: Data curation, Writing – review & editing. YZhan: Investigation, Writing – review & editing. LP: Investigation, Writing – review & editing. CD: Investigation, Writing – review & editing. ZZ: Methodology, Writing – review & editing. LW: Methodology, Writing – review & editing. HX: Methodology, Writing – review & editing. MZ: Methodology, Supervision, Validation, Writing – review & editing. YZhao: Methodology, Supervision, Validation, Writing – review & editing.
